# Comparison of Incidences of Intravascular Injection between Medial and Lateral Side Approaches during Traditional S1 Transforaminal Epidural Steroid Injection

**DOI:** 10.1155/2017/6426802

**Published:** 2017-04-13

**Authors:** Sang Jun Park, Shin Hyung Kim, Seon Ju Kim, Duck Mi Yoon, Kyung Bong Yoon

**Affiliations:** Department of Anesthesiology and Pain Medicine, Anesthesia and Pain Research Institute, Yonsei University College of Medicine, Seoul, Republic of Korea

## Abstract

*Purpose*. Intravascular injection rates are higher during traditional S1 transforaminal epidural steroid injection (TFESI) compared with lumbar transforaminal injection. We compared the incidences of intravascular injection between the medial and lateral approaches to the S1 foramen during S1 TFESI.* Materials and Methods*. A total of 139 patients underwent one or more TFESIs (170 total injections). The patients received S1 TFESI by either medial or lateral side of S1 foramen under fluoroscopic anteroposterior view using digital subtraction method. The intravascular injection rates, epidural spread patterns, and contrast volumes required to reach the superior aspect of the L5-S1 intervertebral disc (SIVD) were compared between groups.* Results*. Intravascular injection rates during S1 TFESI were significantly lower in the medial approach compared with the lateral approach patients (4.9% versus 38.6%, resp., *P* < 0.001). The medial approach group had more epidural spread to the L5-S1 SIVD than the lateral group (82.1% versus 58.8%, resp.); lower contrast volume amounts were required to extend the L5-S1 SIVD (1.46 ± 0.48 versus 1.90 ± 0.62, resp.).* Conclusion*. During S1 TFESI, approaching the needle towards the medial part of the S1 foramen may reduce intravascular injection risk.

## 1. Introduction

Lumbosacral transforaminal epidural steroid injection (TFESI) is an important tool for the nonsurgical management of lumbosacral radiculopathy [1]. However, the needle may commonly be misplaced during these procedures, and incorrect placement of the needle into the spinal vasculature increases the risk of complications and decreases efficacy. The incidence of intravascular injection during TFESI is higher for sacral spine (16.5% to 27.8%) compared with lumbar area (6.1% to 17.7%) injections [2–4].

Traditionally, S1 TFESI has been performed using an anteroposterior (AP) view. In 2007, an oblique view technique using the S1 “Scotty dog” was introduced as an alternative approach by Fish et al. [5]. This approach reduces procedure length and radiation exposure, especially when simultaneous L5 and S1 transforaminal epidural injections are performed. Fish and coauthors also proposed that misplacement of the needle tip anterior to the ventral foramen could be avoided using this approach. Our previous study revealed that, compared with the AP view approach, another advantage of the Scotty dog is the reduction of the intravascular injection rate (29% versus 11%, resp.) [6].

During the Scotty dog approach, the block needle arrives at the sacral epidural space at an inward angle oblique to the sagittal plane. Therefore, the final needle tip location is at the more medial part of the S1 foramen. We hypothesized that while performing the S1 TFESI in the AP view, locating the needle at the medial side of S1 foramen might decrease the incidence of intravascular injection, similar to the Scotty dog approach.

The results of previous studies have indicated that because the vascular contrast pattern appears and disappears quickly during TFESI, live fluoroscopy should be used to observe the dynamic contrast flow [7]. Digital subtraction angiography (DSA) is also useful for interventional spine fluoroscopy procedures. DSA is very effective when used to increase the contrast between vascular structures and adjacent soft tissue and bone [8, 9].

The main objective of this study was to compare the values for incidence of intravascular injection for the medial side versus the lateral side approaches to the S1 foramen during S1 TFESI using live fluoroscopy and DSA. The patterns of the epidural spread of the contrast medium were also compared between the medial and lateral approaches, and contrast volumes required for therapeutic S1 TFESI were identified.

## 2. Methods

### 2.1. Study Population and Randomization

The institutional review board approved the protocol for this randomized prospective clinical trial (reference number 4-2016-0442; ClinicalTrials.gov reference number NCT02867046). Before enrollment in the study, informed written consent was obtained from each patient who received S1 TFESI (139 patients, 20–80 years of age). The patients were enrolled from our outpatient department for pain management between 15 August 2016 and 31 December 2016. Only patients scheduled for an S1 TFESI procedure were included in the study population. A patient was not included in the study if any of the following were present: pregnancy, anatomical sacral abnormality (lumbarization or sacralization), known or suspected coagulopathy, systemic infection, any active injection site infection, or a history of allergy to TFESI injectates (e.g., contrast media, local anesthetics, or corticosteroids). Because of an increased risk of postinjection contrast-induced nephropathy, an estimated glomerular filtration rate < 45 mL/min/1.73/m^2^ was also an exclusion condition.

**Figure 1:**
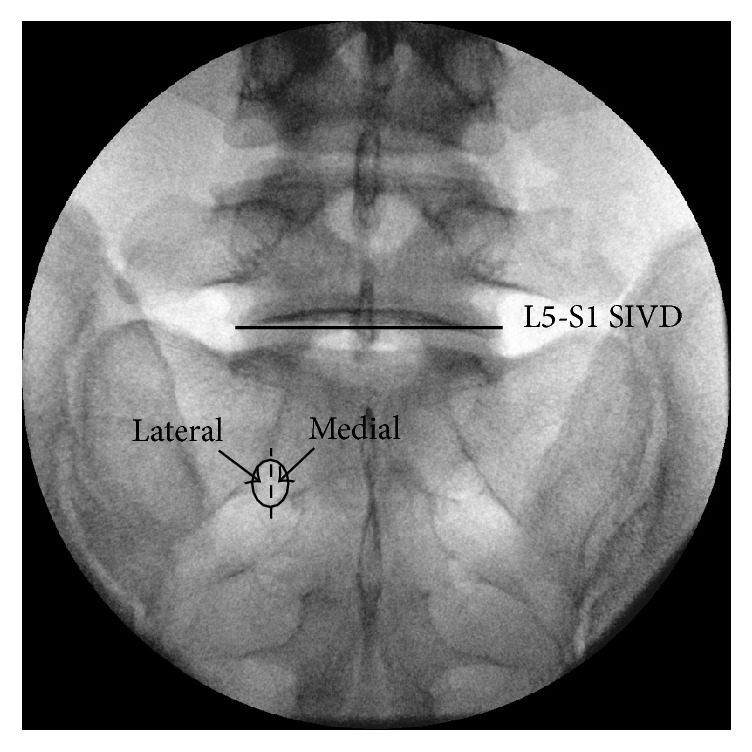
Needle tip target for the medial approach and the lateral approach. Posterior S1 neural foramen (circle). L5-S1 SIVD, superior aspect of the L5-S1 intervertebral disc (line).

Each patient was assigned to the medial or to the lateral approach group using a computer-generated randomization protocol. The S1 TFESI was then given to each patient based on group assignment (Figure 1). The S1 site injection was the first injection given if the patient also received TFESIs at other levels of the spine.

### 2.2. Transforaminal Epidural Injections in S1 and Clinical Data Measures

**Figure 2:**
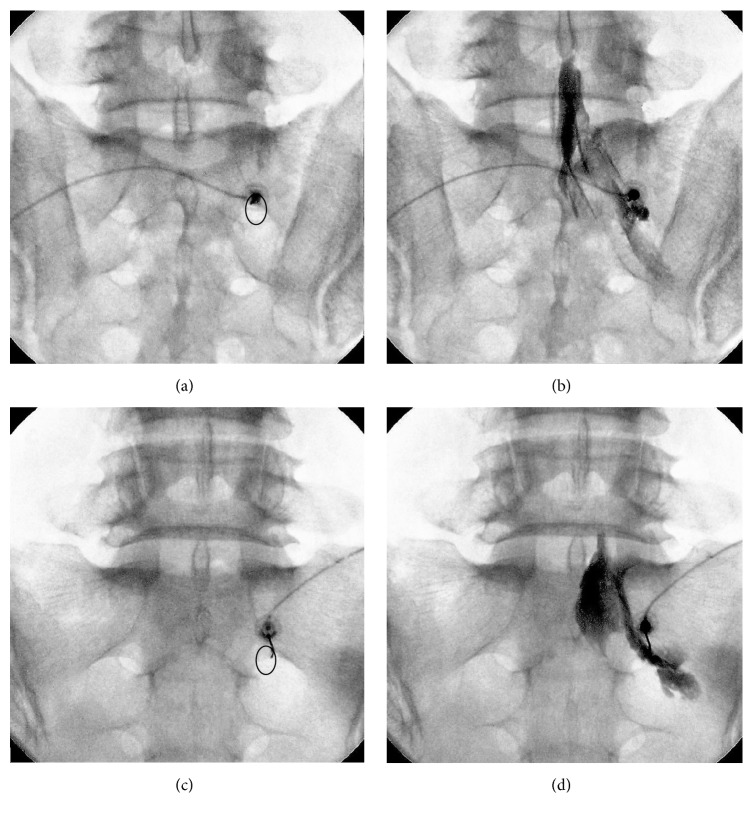
Spinal landmarks and epidural contrast spread patterns viewed using fluoroscopy during S1 transforaminal epidural steroid injection, posterior S1 neural foramen (circle); (a) and (b), medial approach to the S1 foramen in anteroposterior (AP) view, note the spread of medium into the epidural space; (c) and (d), lateral approach to the S1 foramen in AP view.

Fluoroscopic guidance (Siemens Arcadis Varic, Siemens Aktiengesellschaft, Frankfurt, Germany) was used to direct the TFESI at the S1 neural foramen. Patient placement included a prone position, a pillow placed under the lower abdomen, and a sterile drape. A slight cephalad-caudad tilt (image intensifier caudad) was used to maximize the fluoroscopic anatomy opening of the neuroforamen in the AP view (Figure 2). After the skin was infiltrated using 1% lidocaine, a spinal needle (22 G, 8 cm Quincke) was inserted into the medial or lateral side of the S1 neuroforamen using intermittent fluoroscopic guidance. Final advancement was verified using fluoroscopic lateral and AP views. The needle was then attached to an extension tube, and a syringe (5 mL) was connected to the opposite end of the tube. A blood aspiration test was then performed. If it was negative, 1 mL contrast medium was slowly injected (0.1 ml/sec); DSA was used to check for intravascular injection. Appearance and immediate disappearance of a contrast medium snake-flow spread pattern indicated that intravascular injection had occurred. At occurrence, each spread pattern was assigned to one of three categories (i.e., epidural only, epidural and vascular, and vascular only). If vascular spread of the contrast medium was observed, the needle was relocated and lack of vascular uptake was confirmed. If no vascular flow was observed, real-time fluoroscopy was used to guide continued injection of the contrast medium until a total volume of 3 ml was given. The volume of contrast medium required to reach L5-S1 SIVD (Figure 2) was recorded [10]. A lidocaine injection (3 ml, 0.5% with 5 mg dexamethasone disodium phosphate) was given at the end of the procedure.

Three experienced physicians participated in this study. The physician who performed the procedure had experience with C-arm fluoroscopy-guided injections in more than 2000 cases. The vascular spread of contrast medium was confirmed by the other physicians who were not performing the procedure. These physicians also checked the volumes of contrast medium required to reach the specified landmarks during live fluoroscopy.

Data on patient and procedure characteristics were recorded for each patient (e.g., diagnosis, age, sex, body mass index (BMI), side of the procedure, lumbosacral spine surgery history including L5-S1 level surgeries, and underlying disease).

### 2.3. Statistical Analysis

The results of our previous study revealed a 29% incidence of intravascular injection for cases of approach to the S1 foramen in AP view [6]. We considered a 60% decrease in intravascular injection rate in the medial approach to the S1 foramen during S1 TFESI to be clinically relevant. Our statistical power analysis results indicated a required sample size of 85 for each group (*α*-error = 0.05, power = 80%, and dropout rate = 10%). All results were expressed as mean ± standard deviation or median (interquartile range) values or as number of patients. The Shapiro-Wilk test was used to determine whether the data were consistent with a normal distribution. Demographic and clinical data were compared between the two groups using the* t*-test or Chi-square test as appropriate. Multivariate logistic regression analysis was used to assess possible factors (e.g., age, sex, diagnosis, BMI, and spine surgery history). The Statistical Package for the Social Sciences 23 (SPSS Inc., Chicago, IL, USA) application was used for all analyses. A result with a *P* value < 0.05 was considered to be statistically significant.

## 3. Results

**Figure 3:**
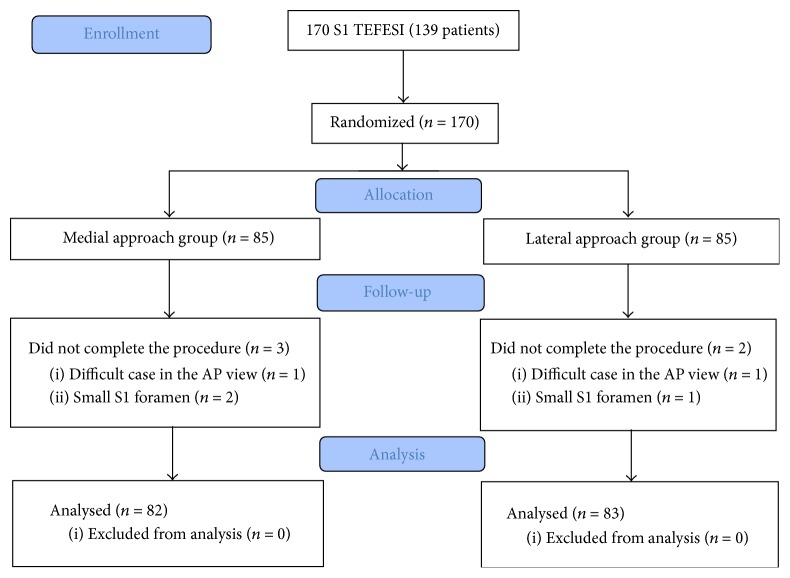
CONSORT flow diagram.

Data from 170 S1 TFESIs performed on 139 patients (31 bilateral injections) were analyzed. During the procedures, the oblique view instead of the AP view was used for one patient from each group because of the difficulty locating the foramen. For two cases in the medial group and one case in the lateral group, it was impossible to distinguish the side of the S1 foramen because of the small size of the posterior S1 foramen. These five patients were excluded from the study population; data from a total of 82 medial group and 83 lateral group cases were analyzed (Figure 3). The results for patients' characteristics and baseline clinical data are presented in Table 1.

The results obtained using DSA with contrast confirmation indicated that the overall intravascular injection rate was 4.9% (4/82) in the medial group and 38.6% (32/83) in the lateral group (Table 2). The values for incidence of simultaneous epidural and vascular injection during S1 TFESI were 100% (4/4) in the medial approach group and 41% (13/32) in the lateral approach group, among the cases that included vascular injection during the procedure.

The results for the epidural spread patterns of contrast medium and the respective volumes of contrast medium required to reach a specific epidural spread pattern landmark are presented in Table 3. Analysis of only epidural spread cases revealed that a higher percentage of medial group cases reached the L5-S1 SIVD, compared with the lateral group (82.1% versus 58.8%, resp.). The medial approach group patients required significantly lower contrast medium volumes to extend to the L5-S1 SIVD, compared with the lateral approach group patients (1.46 ± 0.48 versus 1.90 ± 0.62, resp.).

The results of the multivariate logistic regression analysis are presented in Table 4. Compared with the other variables, a lateral side approach to the S1 foramen was the strongest predictor of intravascular injection during S1 TFESI (odds ratio = 12.874, 95% confidence interval (CI) = 4.063–40.793, *P* < 0.001). Patient's sex, age, BMI, diagnosis, and prior surgery did not have statistically significant association with incidence of intravascular injection (Table 4).

## 4. Discussion

The analysis revealed that, during S1 TFESI, the incidence of intravascular injection in the medial approach group was significantly lower compared with the lateral approach group. Spread of contrast medium to L5-S1 SIVD was also more effective in the medial approach group.

The reason for the lower intravascular uptake incidence in the medial group compared with the lateral group is unclear. However, the same proposal for the lower incidence of intravascular uptake during the S1 Scotty dog approach compared with conventional AP view approach likely contributes to this finding [6]. The decreased vascular uptake incidence using the oblique Scotty dog approach could be explained using Sullivan et al.'s hypothesis [11] regarding the higher incidence of vascular uptake during conventional AP view S1 transforaminal epidural block, compared with the lumbar area. The needle can potentially be positioned along the vessel's path during AP view S1 TFESI, especially when it is in a posterolateral position near the longitudinal vein (part of the posterior internal vertebral venous plexus). Therefore, we speculated previously that, by adopting the oblique Scotty dog approach at the S1 level (approach similar to the lumbar level approach), the rate of intravascular uptake rate would be reduced similarly to the lumbar level by avoiding the posterolaterally located longitudinal veins. During the Scotty dog approach, the block needle arrives at the sacral epidural space at an inward angle oblique to the sagittal plane, so the needle tip will be located at the more medial part of the S1 foramen. Therefore, we hypothesized that while performing an AP view S1 TFESI, locating the needle at the medial side of the S1 foramen might decrease the incidence of intravascular injection in a manner similar to that of the Scotty dog approach. The lower incidence of vascular uptake in the medial approach group patients may be explained by the lower possibility of contact with the posterolateral longitudinal veins.

The reason that contrast medium spread was more effective in the medial group remains to be determined. Epidural spread after S1 TFESI may not be affected by only the needle approach method used, because epidural spread patterns are affected by many variables (e.g., variations in anatomy such as foramen size and formation of postsurgical adhesions, coexisting spinal pathology such as disc pathology and spinal stenosis, and the medical history of the patient). Furthermore, significance of the injectate spread to the disc pathology level cannot be drawn by this observation only.

We encountered some technical problems while performing this study. In some cases, we could not distinguish the medial side from the lateral side because of small foramen size. Occasional minute hemorrhage occurred when the needle contacted unrecognized bony structures (e.g., osteophytes) because the needle approached the edge of the foramen [12]. We did not record data for paresthesia caused by nerve contact, but it was not a problem in either group. The needle was carefully guided as its tip passed through the posterior foramen.

There were some limitations of this study. During the procedure, the physician performing the procedure and the physicians viewing the epidurogram could not be blinded to the side (i.e., medial versus lateral) of the needle approach. Because we did not investigate postprocedure improvement in symptoms such as pain reduction, the results of this study did not provide information on clinical outcomes.

## 5. Conclusion

Although the S1 Scotty dog approach has some advantages, including a lower incidence of vascular uptake, conventional AP approaches are frequently used because of technical familiarity or because sometimes the S1 Scotty dog cannot be clearly seen. This study revealed that when the AP approach is used, introducing the needle at the medial side of the S1 foramen will result in a decreased incidence of intravascular uptake and more effective spread of the contrast medium.

## Figures and Tables

**Table 1:** Results for characteristics of patients and baseline clinical variables.

	Group M (*n* = 82)	Group L (*n* = 83)	*P* value
Female	40 (48.8%)	49 (59%)	0.186
Age, years	63.63 ± 11.67	62.07 ± 15.69	0.470
BMI, kg/m^2^	24.32 ± 2.57	23.93 ± 2.39	0.317
Side			
Right	41 (50%)	35 (42.2%)	0.313
Left	41 (50%)	48 (57.8%)	0.313
Diagnosis			
Spinal stenosis	49 (59.8%)	44 (53.0%)	0.382
HLD	15 (18.3%)	22 (26.5%)	0.206
FBSS	5 (6.1%)	10 (10.8%)	0.274
Spinal stenosis and HLD	11 (13.4%)	7 (8.4%)	0.305
Previous surgery	16 (19.5%)	17 (20.5%)	0.876

Values are expressed as the mean ± standard deviation or number of patients (%). Group M, medial approach group; Group L, lateral approach group; HLD, herniated lumbar disc L5-S1; FBSS, failed back surgery syndrome.

**Table 2:** Incidence of intravascular injection during S1 transforaminal epidural steroid injection.

	Group M (*n* = 82)	Group L (*n* = 83)	*P* value
Epidural only, *n*	78 (95.1%)	51 (61.4%)	<0.001^*∗*^
All vascular, *n*	4 (4.9%)	32 (38.6%)	<0.001^*∗*^
Epidural and vascular	4 (4.9%)	13 (15.7%)	0.023^*∗*^
Vascular only	0	19 (22.9%)	<0.001^*∗*^

Values are expressed as the mean ± standard deviation or number of patients (%). Group M, medial approach group; Group L, lateral approach group; SIVD, superior aspect of the L5-S1 intervertebral disc. ^*∗*^*P* value < 0.05.

**Table 3:** Numbers of patients for whom contrast medium reached an L5-S1 SIVD in epidural only group and volume of contrast medium required for spread to L5-S1 SIVD during S1 transforaminal epidural steroid injection.

	Group M (*n* = 78)	Group L (*n* = 51)	*P* value
Spread to L5-S1 SIVD, *n*	64 (82.1%)	30 (58.8%)	0.004^*∗*^
Volume of contrast medium, mL	1.46 ± 0.48	1.90 ± 0.62	0.001^*∗*^

Values are expressed as the mean ± standard deviation or number of patients (%). Group M, medial approach group; Group L, lateral approach group; SIVD, superior aspect of the L5-S1 intervertebral disc. ^*∗*^*P* value < 0.05.

**Table 4:** Logistic regression analysis of potential factors associated with intravascular injection during S1 transforaminal epidural steroid injection.

	OR (95% CI)	*P* value
Lateral approach	12.874 (4.063–40.793)	<0.001^*∗*^
Female	1.613 (0.659–3.950)	0.296
Age	1.021 (0.982–1.061)	0.300
BMI	0.911 (0.766–1.083)	0.289
Diagnosis		
Spinal stenosis	0.136 (0.007–2.638)	0.188
HLD	0.322 (0.019–5.540)	0.435
FBSS	0.077 (0.002–2.416)	0.145
Spinal stenosis and HLD	0.086 (0.003–2.336)	0.145
Previous surgery	2.422 (0.646–9.072)	0.189

Values are number (95% CI). HLD, herniated lumbar disc L5-S1; FBSS, failed back surgery syndrome. ^*∗*^*P* value < 0.05.
